# Adenosine Receptor Reserve and Long-Term Potentiation: Unconventional Adaptive Mechanisms in Cardiovascular Diseases?

**DOI:** 10.3390/ijms22147584

**Published:** 2021-07-15

**Authors:** Régis Guieu, Michele Brignole, Jean Claude Deharo, Pierre Deharo, Giovanna Mottola, Antonella Groppelli, Franck Paganelli, Jean Ruf

**Affiliations:** 1 Center for CardioVascular and Nutrition Research, INSERM, INRAE, Aix-Marseille University, 13005 Marseille, France; jean-claude.deharo@ap-hm.fr (J.C.D.); pierre.deharo@ap-hm.fr (P.D.); giovanna.mottola@univ-amu.fr (G.M.); franck.paganelli@ap-hm.fr (F.P.); jean.ruf@univ-amu.fr (J.R.); 2 Laboratory of Biochemistry, Assistance Publique des Hopitaux, 13005 Marseille, France; 3 IRCCS, Istituto Auxologico Italiano, Ospedale San Luca, 13000 Milan, Italy; mbrignole@outlook.it; 4 Department of Cardiology, CHU Timone, 13005 Marseille, France; 5 Cardiology Department, Hopital Nord, 13005 Marseille, France; antonella.groppelli@auxologico.it; 6 Department of Cardiovascular, Neural and Metabolic Sciences, Faint & Fall Programme, IRCCS Istituto Auxologico Italiano, San Luca Hospital, 13000 Milan, Italy

**Keywords:** adenosine receptor reserve, arrhythmia, coronary artery disease

## Abstract

While the concept of a receptor reserve (spare receptors) is old, their presence on human cells as an adaptive mechanism in cardiovascular disease is a new suggestion. The presence of spare receptors is suspected when the activation of a weak fraction of receptors leads to maximal biological effects, in other words, when the half-maximal effective concentration (EC_50_) for a biological effect (cAMP production, for example) is lower than the affinity (K_D_) of the ligand for a receptor. Adenosine is an ATP derivative that strongly impacts the cardiovascular system via its four membrane receptors, named A_1_R, A_2A_R, A_2B_R, and A_3_R, with the A_1_R being more particularly involved in heart rhythm, while the A_2A_R controls vasodilation. After a general description of the tools necessary to explore the presence of spare receptors, this review focuses on the consequences of the presence of spare adenosine receptors in cardiovascular physiopathology. Finally, the role of the adenosinergic system in the long-term potentiation and its possible consequences on the physiopathology are also mentioned.

## 1. Introduction

Clark’s receptor theory [1] describes the relationship between a ligand and its receptor as a linear response, suggesting that the maximal receptor response to a drug or an endogenous ligand is equal to the maximal tissue response. This theory also assumes that drugs interact with receptors in a reversible manner to produce a conformational change in the receptor, and that the interaction between ligand and receptor obeys the law of mass action. In addition, the ligand binding to the receptor determines the quantitative relationship between ligand concentration and biological effects.

**Figure 1:**
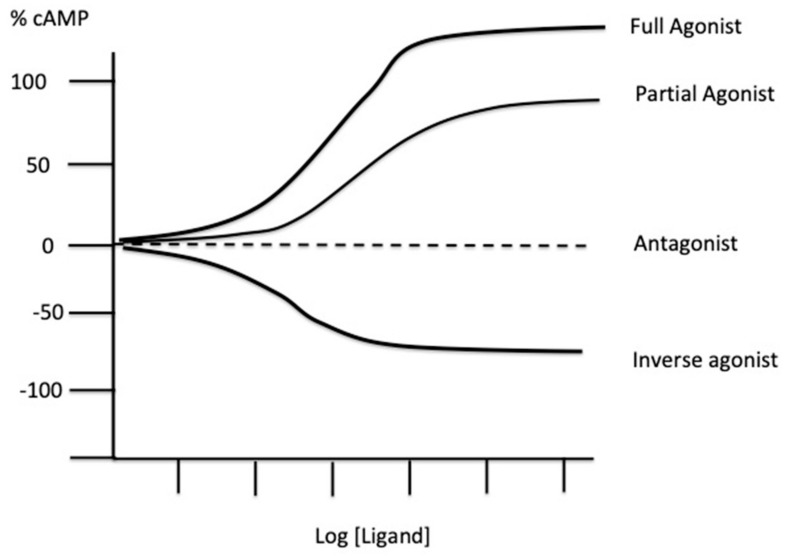
Functional consequences of the binding of a ligand (exogenous or endogenous) on its receptor. Agonists activate receptors, while classic antagonists occupy the agonist binding pocket and block agonist effects. Partial agonists have a lower efficiency than full agonists do. Inverse agonists stabilize the receptor in an inactivated state and decrease basal effects in a dose-dependent manner. Basal response can be expressed as % cAMP production. From [6], modified.

In 1954, Stephenson [2] proposed the concept of stimulus and efficiency. The agonist stimulates the receptor system (while the antagonist inhibits it) to produce a tissue response, and there may be a nonlinear relationship between the stimulation and the response (i.e., between the ligand concentration and biological effects) [2]. The concept of efficiency was defined as the unit stimulus per occupied receptor. The reciprocal affinity between the ligand and its receptor determines the selectivity, while competition between molecules for the same receptors determines the notion of full or partial agonist, antagonist, and inverse agonist. Note that this concept implies the possibility of a basal activity in the absence of an agonist ([3,4] and Figure 1). Three distinct processes have been proposed that characterize ligand–receptor interaction: (i) receptor binding, defined by the equilibrium dissociation constant (K_D_), used to determine affinity; (ii) receptor activation that introduces the notion of efficiency; and (iii) post-activation transduction signal that is mostly characterized by a signal amplification [5]. In general, receptor models are two-state models corresponding to the occupied and active receptor form, which do not fully correspond. However, activation of the receptor can be maintained after ligand binding to its receptor by an induced fit mechanism. Thus, the receptors can be active or inactive regardless of their occupation by the ligand. The free and ligand bound states of the receptor are considered to be in equilibrium [5].

Using the histamine receptor model, and an irreversible antagonist, Nickerson et al. [7], however, demonstrated that the receptor occupancy is not the limiting factor for tissue activation and that about 1% of the histamine receptors’ activation is sufficient to produce a maximal tissue response. The notion that full agonists can achieve their maximal effects without occupying all available receptors on the cell surface was thus established and the notion of a receptor reserve was born.

The presence of a receptor reserve (spare receptors) supposes a strong signal amplification and is dependent upon the agonist nature [8,9]. The receptor reserve depends upon three factors: (i) the agonist efficiency (i.e., concentration–effects relationship); (ii) the different functional states of the receptor and the tissue-dependent signal amplification [10]. In most cases, in the absence of spare receptors, the affinity constant value (K_D_) is close to [11] or lower than the half-maximal value of the biological effects (EC_50_) [12,13], depending on the nature of the receptor and ligand system.

From a pharmacological point of view, the presence of spare receptors for an agonist is suspected when maximal or near-activation occurs during partial occupation of the receptors [14], in other words, when the maximal biological effect (assessed, for example, by the cAMP production) is obtained at a lower agonist concentration than that required for the occupancy of all the receptors (Bmax). Using conventional parameters, the presence of a receptor reserve is suspected when the half-maximal biological effect value (EC_50_) is lower than the agonist affinity constant for the receptor (K_D_). This can also be assessed by a low EC_50_/K_D_ ratio [9,15]. Conversely, the Ki/EC_50_ ratio can also be achieved when using an antagonist [16]. The presence of spare receptors suggests a strong signal amplification [5]. This amplification occurs via a downstream cascade of saturable functions provided mainly by the second messenger. This process appears crucial such that a cell, a cell group, a tissue, or an organ can continue to be activated despite a shortage of ligands or receptors. The most common example is the stimulation of beta receptors by epinephrine, where a half-maximal increase in muscle contractility occurs with only a 1 to 3% receptor occupancy in rats and 10 to 20% in humans [17]. As a result of signal amplification, epinephrine at a concentration as low as 10^−10^ M promotes liver glycogenolysis via the cAMP production in the range of 10^−6^ M (suggesting a 10,000-fold amplification) and releases sufficient physiological glucose in blood [18].

## 2. Experimental Tools to Evaluate the Presence of Spare Receptors

The presence of a receptor reserve was identified first for histamine receptors [7] and then for alpha and beta adrenergic [17,19], muscarinic [20], dopamine [21], adenosine [15,22], serotonin [23], and T cell receptors [24,25]. To determine the presence of a receptor reserve, it is necessary to assess the relationship between receptor occupancy and biological response. This was first carried out using the method developed by Furchgott and Bursztyn [26] based on the calculation of the equilibrium dissociation constant and the fraction of occupancy of receptors by an agonist before and after irreversible inactivation of a fraction of the receptor population.

This method implies the use of an irreversible ligand, the term “irreversible” meaning that the ligand binds to the receptor in an irreversible manner, at least for the length of the experimental procedure [9,15]. The ligand must not leave one receptor to bind to another one, at least during the test. The presence of spare receptors has been demonstrated using irreversible organic agonists [22] or antagonists [15,20,21]. Chemoreactive ligands are useful probes to study ligand–receptor interactions. These probes are usually composed of a pharmacophore associated with a reactive moiety, which allows a covalent link between ligand and receptors [15,27,28].

More recently, a monoclonal antibody with agonist properties was developed, which makes it possible to synchronously evaluate the biological effects (i.e., cAMP production) and K_D_ for the G-coupled A_2A_R [29]. Using this kind of tool, it is possible to directly assess the EC_50_/K_D_ ratio in a same binding test [12,30,31]. 

## 3. Spare Receptors in the Adenosinergic System

Adenosine via its four G-coupled membrane receptors named A_1_R, A_2A_R, A_2B_R, and A_3_R impacts several systems, including mainly the immune, the cardiovascular, and the nervous systems [32]. A_1_R and A_3_R activation leads to cAMP production inhibition, while the activation of A_2A_R and A_2B_R leads to cAMP production in target cells. The dominant effects of adenosine in the vascular system are vasodilation through the activation of A_2A_R [33,34] and A_2B_R [35]. The vasodilation occurs through the production of cAMP [33] and the activation of Kv channels, K_ATP_ channels, and the NO pathway [36,37]. Vasodilation through A_2A_ and A_2B_R activation also occurs in the coronary arteries [15,38].

Conversely, the activation of A_1_R leads to vasoconstriction in the aorta and mesenteric arteries [39,40]. A_1_R is also expressed in the renal microcirculation [41]. Their activation leads to vasoconstriction through cAMP production inhibition and Gi-dependent phospholipase C activation [42]. The activation of A_1_R also potentiates angiotensin II [43] and norepinephrine [41]-induced vasoconstriction. Finally, the activation of A_1_R also leads to the modulation of tubuloglomerular feedback [44], which is an important mechanism in control of renal hemodynamics and blood pressure via the regulation of salt-water balance.

Schematically, in the heart, the activation of A_1_R leads to bradycardia, sinus arrest, and sometimes, atrioventricular block [45], while the activation of A_2A_R and A_2B_R leads to vasodilation, particularly (but not only) in the coronary system [15,38]. In the nervous system, there is a large presence of adenosine receptors in the brain on both neurons and glial cells [46]. The adenosinergic system influences sleep, arousal, cognition, memory, and neuronal damage [47]. Adenosine also modulates neurodegenerative diseases such as Parkinson’s or Alzheimer’s disease [47]. Adenosine also has antiepileptic [48,49] and antineuropathic pain properties [50]. Adenosine receptors are fully present on immune cells where they control the inflammatory response [51,52]. Interestingly, T-cell receptors (TCR) are the archetype of the spare receptor model, because their presence is required for responses to low concentrations of agonists [24]. Thus, agonists are unaffected by a 90% reduction in TCR level, while proliferation to weak agonists is significantly inhibited when TCR expression is reduced by 40% [24].

Determining the presence of spare adenosine receptors is challenging for molecules having a very short half-life. In this context, a mathematical model of the agonist–receptor interaction allowed the identification and quantification of spare adenosine receptors [10]. Peripheral blood mononuclear cells (PBMC) are used to evaluate the production and function of adenosine receptors in the cardiovascular system as the behavior of the adenosine receptors on PBMC mirror the behavior of these receptors in the heart [53], coronary arteries [54], and peripheral arteries [13]. Thus, the expression level and the cAMP production in PBMC are the same as in the cardiovascular tissues.

The presence of spare A_1_R has been determined in a guinea pig heart using an irreversible A_1_R agonist [22]. In this study, the relationship between irreversible A_1_R binding and the inhibition of cAMP production in a cell culture revealed a large receptor reserve of about 64%. However, the receptor reserve fraction was lower (10–20%) on His bundle tissue, suggesting that the evaluation of spare receptors also depends upon the nature of agonists, the biological effects, and the nature of target tissues. Srinivas et al. [55] using guinea pig atrial myocytes showed that A_1_R stimulation by adenosine activates inwardly rectifying K+ channels (I_Kado_) and inhibits isoproterenol-stimulated L-type Ca++ channels with an EC_50_ more than 10 times higher for the first than for the second current effects. Thus, the half-maximal activation of I_Kado_ required 40% of receptor occupancy, while the occupancy of only 4% of receptors led to half-maximal inhibitory effects on isoproterenol-stimulated L-Type calcium channels, suggesting the presence of a large spare A_1_R population. 

The presence of spare A_2A_R was first demonstrated by Shryock et al. [15] in an isolated and perfused guinea pig heart model. These authors used an irreversible antagonist (SCH58262) to inactivate receptors and to reduce the response to agonists. Then, they used two distinct organic agonists: CGS21680, CCPA, in addition to adenosine. They found that the EC_50_ for increasing coronary conductance was 70-, 11-, and 21-fold lower than those of the affinity constant (K_A_), respectively, for the three agonists. In other words, the half-maximal response to these agonists required the occupation of 1%, 9%, and 5%, respectively, of the A_2A_R total number. These results demonstrate the presence of a large part of A_2A_R reserve in the guinea pig heart. 

## 4. Biological Consequences of the Presence of Spare Receptors

One implication of the presence of spare receptors is the increase in the number of ligands for a given response, which allows cell activation by weak ligands. The presence of spare receptors also masks a partial agonist activity of drugs. This was demonstrated using a transgenic cell line that stably expresses the 5-hydroxytryptamine receptor [23]. In this model, EC_50_ values (evaluated by the cAMP production) for agonists were lower than expected from the Ki values. When spare receptors are present, the concentration of antagonist necessary to have biological effects should be very high. For example, if only 5% of the receptors occupied by the agonist are sufficient to produce maximum biological effects, the antagonist must occupy more than 95% of the free receptors to displace the agonist. This requires a high antagonist concentration.

In a guinea pig heart, the exogenous concentration of adenosine that is required to produce half-maximal vasodilation in coronary arteries via A_2A_R activation is ten-fold lower than that necessary to cause electrophysiological (i.e., negative dromotropic or chronotropic) effects [56]. Thus, the presence of spare receptors makes it possible to promote one biological action rather than another depending on the available concentrations of adenosine. Another consequence of the presence of spare A_2A_R is that the release of adenosine can increase coronary blood flow without affecting systemic blood pressure [57,58]. This latter effect suggests that the presence of spare receptors is tissue-dependent. 

## 5. Possible Clinical Consequences of the Presence of Spare Receptors

### 5.1. Spare Receptors and Coronary Artery Disease (CAD)

Coronary artery disease (CAD) is a cardiovascular disease that is the leading cause of death worldwide [59]. CAD is an atherosclerotic disease that affects the coronary arteries, with an important role played by inflammation and manifesting as stable or unstable angina, myocardial infarction (MI), and sometimes sudden death. Alteration in coronary blood flow (CBF) is the main consequence of atherosclerosis in CAD patients. While it is likely that adenosine is poorly implicated in the regulation of CBF under resting conditions, the contribution of adenosine and its receptors plays a major role in the CBF adaptation to an increase in oxygen need that occurs during exercise or when a drop in oxygen supply occurs such as in hypoxia or ischemia [60,61]. The coronary vasodilation occurs mainly via the activation of A_2A_R and A_2B_R [15,62,63].

**Figure 2:**
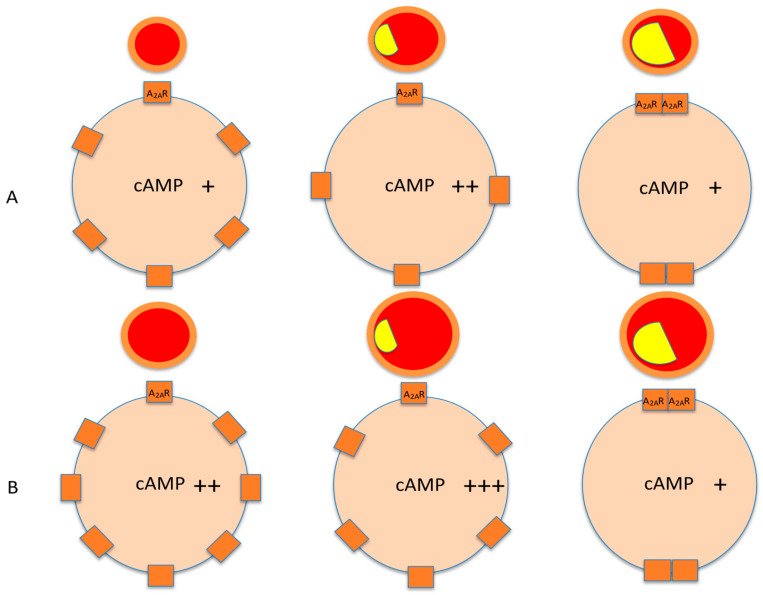
Schematic representation of the pharmacological profile evolution of A_2A_ adenosine receptors (A_2A_R) during progressive atherosclerosis coronary obstruction at rest (**A**) and during exercise test (**B**). Endogenous adenosine release from cardiac myocytes by global myocardial ischemia dilates coronary arteries through cAMP pathway. Adenosine binds to A2 receptors on coronary smooth muscle cells (CoSMCs), increasing cyclic adenosine monophosphate (cAMP), which opens calcium-activated potassium channels. Orange circles represent the arterial wall with CoSMCs. Red circles represent the lumen of the coronary arteries, which is progressively obstructed by atherosclerosis (yellow) and vasodilated by cAMP. During exercise stress testing (**B**), the left panel (healthy artery) shows that cAMP production is higher than that at rest (left panel A) because adenosine is released during exercise and is associated with an increase in coronary vasodilation induced by cAMP (cAMP production and coronary dilation being correlated). Regarding the middle panel (CAD with stenosis), cAMP production is increasing to compensate partial obstruction at rest with more cAMP secretion during exercise. The increase in cAMP production remains effective during effort. In the right panel (CAD with ischemia), the spare receptor phenomenon appears. cAMP production is noneffective because of a small number of oligomerized A_2A_R. Note that in the middle panels at rest (**A**) and during effort (**B**), cAMP increases, whereas a lower number of A_2A_R are expressed on the cells, which can constitute a signal for oligomerization.

As previously described, the presence of spare receptors allows the receptors of the target cells to be activated in spite of a low extracellular adenosine concentration. In addition, their presence makes it possible to promote one biological action rather than another depending on the extracellular concentration of adenosine and the number of available receptors. This is particularly true for the coronary arteries. Thus, in patients with CAD, the presence of spare A_2A_R was associated with a low production of A_2A_R both on PBMC and coronary arteries [12,30,31,54]. While the presence of spare receptors was reported in coronary arteries from healthy animals [15], the presence of adenosine A_2A_R in humans seems associated with cardiovascular diseases. The presence of spare receptors seems to be an adaptive mechanism that allows the target cells to process a signal transduction pathway despite a low adenosine concentration, a low number of available receptors, or both [9]. In CAD patients, the presence of spare receptors has been identified in patients with inducible ischemia, revealed by a low coronary flow fraction reserve [12] or by a positive stress test [30]. While one would expect an increase in A_2A_R production to counteract the decrease in coronary blood flow, surprisingly, a decrease in A_2A_R production in CAD patients with severe stenosis was observed [12,13,30,31,54]. We hypothesize that the first step of the adaptive mechanism in CAD to inducible ischemia is an asymptomatic and transient increase in A_2A_R under chronic adenosine stimulation. In the second step, the high A_2A_R density forces the receptors themselves to oligomerize at the cell surface, a process that would confer the characteristics of spare receptors (EC50 < KD) on A_2A_R. This is in accordance with one of the revisited models from the initial theory, that a single site occupied by an agonist for a group of several receptors connected to an effector may be sufficient to achieve a full effect [63]. Most of the oligomerized A_2A_R could then enter cells to be exported in exosomes as a rescue route [64]. The oligomerized A_2A_Rs remaining on the cell surface constitute the spare A_2A_R identified in CAD patients at high cardiac risk. (see Figure 2). This hypothesis is supported by the fact that spare A_2A_R has been observed in the case of low A_2A_R production and a normal or low range of adenosine plasma concentration [12,13,30,54], but also in the case of high A_2A_R production associated with a very low extracellular adenosine concentration [65,66] in the event of neurocardiogenic syncope (see next paragraph). It seems that some conditions can influence the appearance of spare receptors, especially the conditions that occur during severe ischemia or hypoxia. Indeed, the appearance of spare receptors was observed during a CEM cell culture exposed to a chemical model of hypoxia (unpublished data). This suggests that the decrease in O_2_ (and/or pH, lactate accumulation, or other unknown factors induced by hypoxia) may trigger the appearance of spare receptors.

Finally, the detection of spare A_2A_R on PBMC could be an innovative way to screen CAD patients with severe stenosis (12), or to detect, among the patients to be operated on in vascular surgery, those whose preoperative assessment requires a coronary exploration before anesthesia and surgery [13].

### 5.2. Spare Receptors and Arrhythmia

**Figure 3:**
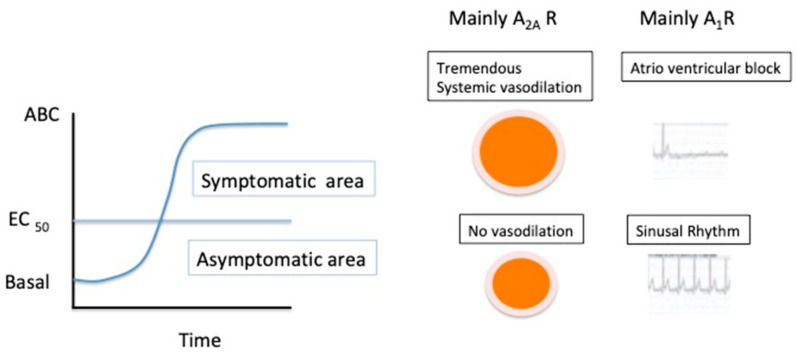
Effects of an increasing adenosine blood concentration (ABC) on the cardiovascular system in patients with neurohumoral syncope associated with low adenosine plasma concentration and spare adenosine receptors.

Abnormalities in the concentration of adenosine blood level and the production of adenosine receptors (especially A_2A_R) evaluated on peripheral blood mononuclear cells have been reported in patients suffering from neurohumoral syncope. The presence of spare A_2A_R has also been associated with certain types of neurocardiogenic syncope [65,66]. In this disease, the adenosinergic system plays a major role [33,66,67,68,69,70,71] as high adenosine plasma concentrations and high A_2A_R productions were measured in the subgroup of patients with vasovagal syncope [65]. Furthermore, the subgroup of neurocardiogenic syncope patients with low adenosine plasma levels are very sensitive to adenosine administration (ATP test) and are clinically characterized by a sudden loss of consciousness in the absence of prodromes before fainting [67,68,69,70,71]. In these patients, the loss of consciousness is mainly due to severe bradycardia (especially atrioventricular block), dramatic vasodilation, or both. Some of the patients have spare A_2A_R associated with a very low concentration in adenosine plasma level [65,66]. The presence of spare receptors in the context of low adenosine plasma concentration could explain the sudden syncope without prodromes. Indeed, under basal conditions, when the plasma adenosine concentration is lower than the EC_50_ value, no effect on the cardiovascular system is observed. However, when the adenosine plasma concentration exceeds the EC_50_ value, the activation of spare receptors induces a sudden maximal cAMP production associated with vasodilation and/or severe bradycardia or AVB (Figure 3).

**Figure 4:**
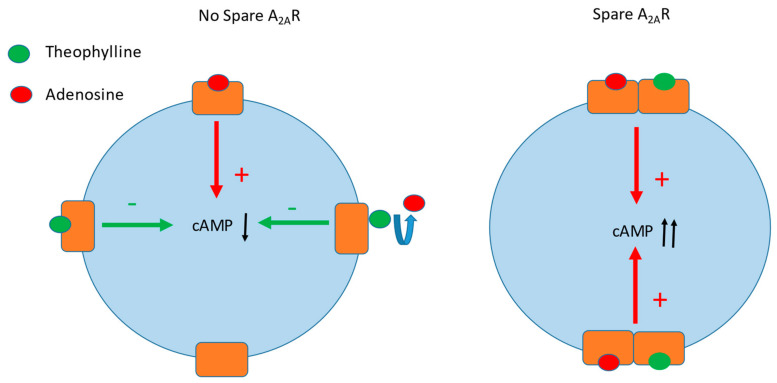
In the absence of an adenosine receptor reserve (spare, **left** panel), theophylline inhibits the production of cAMP following the activation of A_2A_R by adenosine in a dose-dependent manner. In the presence of spare (**right** panel), the activation by adenosine of a small fraction of adenosine receptors leads to a maximal production of cAMP in target cells, rendering ineffective the inhibition of cAMP production by the antagonist (theophylline).

Theophylline, a nonspecific adenosine receptor antagonist, is a useful treatment for syncope patients with low adenosine levels. However, in some cases, theophylline fails to prevent syncopal episodes [72,73]. The presence of spare receptors may explain these cases of treatment failures. Indeed, as explained above, theophylline concentration necessary to counteract adenosine must be very high (Figure 4).

### 5.3. Spare Receptors and Atrial Fibrillation

Adenosine and its receptors are also involved in atrial fibrillation (AF). Indeed, adenosine administration leads to the triggering of an AF episode in susceptible patients [74]. In addition, a higher adenosine plasma level in the left atria has been shown to be associated with AF episodes that normalize after cardioversion [75]. High A_2A_R production in an AF patient’s atria was also reported [76,77]. In particular, an increased adenosine blood level, reduced adenosine deaminase activity, and upregulation of A_2A_R of PBMC have been reported in patients with AF. In addition, a positive and significant correlation between A_2A_R production in the right atrium and in PBMC was observed [77].

The adenosine-induced AF episode is probably due to an increase in the refractory period secondary to the activation of inwardly rectifying potassium channels [78]. In patients undergoing cardiac surgery, perioperative episodes of AF occur in 10 to 50% of cases [79,80]. These episodes are often associated with an increase in adenosine plasma level [81,82].

The effects of caffeine, a nonspecific adenosine receptor antagonist on AF, is controversial. Moderate caffeine consumption seems to have protective effects on arrhythmias; on the contrary, high caffeine consumption seems to be associated with an increased risk of AF [83]. However, the use of caffeine failed to decrease the occurrence of perioperative AF [82]. This last result can be explained by the fact that caffeine increases adenosine plasma levels [84] and A_2A_R production [85] or, alternatively, increases the sensitivity of A_2A_R agonists [86]. In this latter case, due to the presence of spare receptors, the activation of only a small fraction (free caffeine receptors) by adenosine leads to a maximal production of cAMP despite the blockade of a large receptor number by caffeine. Thus, as with syncope, the presence of spare adenosine receptors makes the action of adenosine receptors antagonists uncertain.

### 5.4. Role of Adenosine Receptors in the Induction of Long-Term Potentiation

Apart from the presence of a receptor reserve described above, we cannot end this review without giving an overview of the phenomenon of long-term potentiation (LTP), where the adenosine receptors seem to be strongly involved. A long residence time offers the potential to increase the duration of synaptic activation beyond and sometimes independently to the half-life and to the pharmacokinetic profile of the ligand in the extracellular spaces [87]. The timing of the agonist (or antagonist) response is crucial for the biological activities and for treatment when the ligand has potentially therapeutic effects.

In some cases, in spite of a very short life duration, agonists (or antagonists) may have long lasting effects. Experimentally, LTP has been described as a persistent increase in synaptic strength following high-frequency stimulation of a chemical synapse [88]. It is a phenomenon by which the “memory” of the signal transduction pathway activation (or inhibition) may continue for several minutes or hours to be activated (or inhibited, LTD for long-term synaptic depression) while the activating or inhibiting agent has disappeared. This phenomenon can be experimentally induced by the delivery of burst stimulation [88], but it is likely that this phenomenon occurs spontaneously in the nervous system. Indeed, while LTP is under physiological conditions implicated in memory and learning, in some cases, it can participate in diseases such as seizures [89]. The long-term effects have been attributed to a persistent calcium release following high-frequency stimulation [90]. As adenosine receptors are strongly implicated in the control of calcium release in excitable cells, it is not surprising that the adenosinergic system is involved in the LTP phenomenon.

While the activation of A_2_ adenosine receptor subtypes seems to facilitate LTP, conversely, A_1_R activation has opposite effects [91]. Indeed, LTP is facilitated in the presence of the selective A_1_R antagonist, and is reduced by the adenosine uptake blocker, suggesting that endogenous adenosine exerts a tonic inhibitory role on long-term potentiation, which is mediated through adenosine A_1_R [92]. Interestingly, a brief exposure to hypoxia leads to the inhibition of LTP, via a massive efflux of adenosine that activates pre- and post-synaptic A_1_R [93]. In the accumbens nucleus, LTP appears to be modulated by the A_2A_R pathway, as the level of potentiation was reduced in A_2A_R-deficient mice. This modulation occurs via cAMP-dependent protein kinase [94]. Thus, adenosine acting at the A_2A_R is implicated in events related to LTP induction [95]. Although, it was described in the hippocampus [88], this phenomenon can also occur in autonomic ganglia (called ganglionic LTP; gLTP [96]), when repetitive impulses travel from the central nervous system to the periphery. Experiments in rat sympathetic ganglia suggest similar molecular mechanisms for the expression of gLTP and for hippocampal LTP [96,97].

## 6. Possible Clinical Consequences of LTP

Triggers such as chronic stress and repetitive seizure could cause a lasting increase in sympathetic tone to the cardiovascular system, leading to hypertension and arrhythmia, and, sometimes, sudden cardiac death [97]. LTP has been described for adenosine A_1_R, A_2A_R, and A_2B_R in the supra spinal central nervous system [96], but may also impact the cardiovascular system through the autonomic system. Thus, expression of gLTP in autonomic ganglia may participate in blood pressure disturbance and cardiac arrhythmias [97], and may be a serious risk factor for morbidity and mortality. LTP could participate, through the adenosinergic system, in certain cases by memory effect, in the recurrence of neurohumoral syncope but also to the transition from paroxysmal to permanent fibrillation.

## 7. Conclusions

Spare adenosine receptors appear to be an adaptive mechanism that allows target cells to be regulated in spite of low adenosine concentration, low adenosine receptor production, or both. Further investigations are necessary to know whether their presence is genetically predisposed. Finally, the LTP process seems to involve some pathophysiological processes such as blood pressure disturbance or arrhythmia.

## Data Availability

Not applicable.
